# An Interesting Case of Post-enucleation Retinoblastoma Presenting With Distant Liver and Skeletal Metastases Detected on 18F-Fluorodeoxyglucose Positron Emission Tomography-Computed Tomography

**DOI:** 10.7759/cureus.48249

**Published:** 2023-11-04

**Authors:** Ritwik Wakankar

**Affiliations:** 1 Nuclear Medicine, All India Institute of Medical Sciences, New Delhi, Delhi, IND

**Keywords:** unilateral retinoblastoma, pet-ct, fdg pet-ct, metastatic retinoblastoma, retinoblastoma

## Abstract

Retinoblastomas are the most common primary ocular malignancies in the pediatric population. They are known to undergo metastasis to distant sites like the bone marrow, lymph nodes, and skeleton, but liver metastasis is rare. 18F-fluorodeoxyglucose positron emission tomography-computed tomography is an interesting imaging technique that allows for staging for disease metastasis and plays a crucial role in treatment planning and prognostication of retinoblastoma patients with suspected distant metastasis. The case report highlights this very fact by demonstrating the case of a one-year-old boy with retinoblastoma that had metastasized to the skull bones and even the liver.

## Introduction

Retinoblastoma is the most common intraocular pediatric malignancy in the world, with the majority of the patients being under five years of age at the time of presentation. The five-year survival rates are 95% in developed countries, but retinoblastoma can be very lethal if left untreated. The tumor mostly remains limited to the ocular bulb, with distant metastasis being quite rare. In the event that distant metastasis does occur, it is mostly in the marrow, lymph nodes, or the cortical skeleton, with visceral metastasis being very uncommon [1-3]. Retinoblastoma develops due to a loss-of-function mutation of the retinoblastoma (RB) protein in the retinal progenitor cells, subsequent to inactivating mutations in the RB1 alleles. The disease can be either sporadic or germline in its presentation, and children can have either a unilateral or bilateral presentation [4]. Children who have the germline RB mutation tend to present with bilateral retinoblastomas and are also at an increased risk of subsequent primary malignancies (SPM) later on in life, which include cancers like osteosarcoma, melanoma, leukemia, lung cancer, etc. The incidence of SPM has been linked to the use of external beam radiotherapy and alkylating chemotherapeutic agents [3,5-8].

## Case presentation

**Figure 1:**
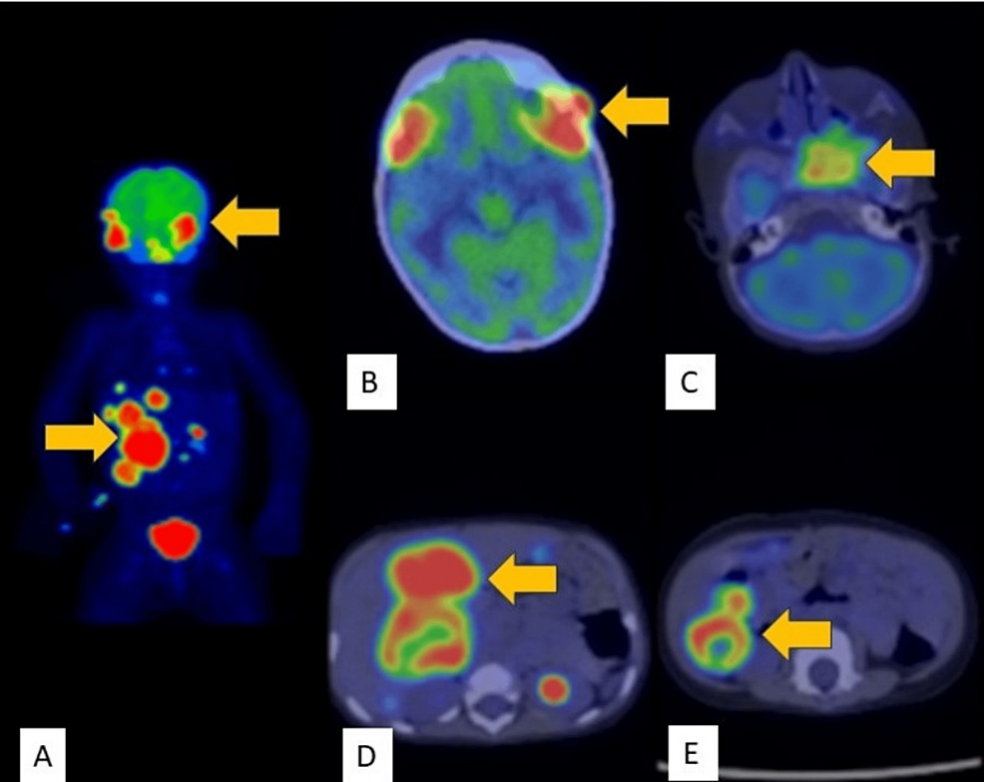
18F-fluorodeoxyglucose positron emission tomography-computed tomography scan of a patient with metastatic retinoblastoma (highlighted with solid yellow arrows). Maximum intensity projection image showing hypermetabolic lesions in the skull and liver (A). Fused axial images demonstrating hypermetabolic lytic lesions in bilateral frontal and sphenoid bones (B, C) with hypermetabolic lesions in the liver (D, E)

A one-year-old boy (height: 70 cm, weight: 8 kg) from a low socio-economic stratum, presented to the pediatric oncology outpatient clinic with complaints of multiple swellings in his scalp as noticed by the parents. The history was negative for head trauma. However, it was significant for a history of retinoblastoma of the left eye, which was diagnosed at the age of two months. The child had undergone an enucleation of the left eye, at a different hospital. Unfortunately, the parents did not have any documentation with them as to the exact nature of the procedure whether any other imaging had been done prior to the enucleation, or if any chemotherapy/radiotherapy had been given to the child. According to them, the child was completely healthy up to two months ago when he first noticed a small swelling on his scalp, which they did not take seriously at the time. However, when the child’s swelling kept on gradually increasing in size and was accompanied by the appearance of other new scalp swellings, they became concerned and decided to get the child evaluated by a local pediatrician, who then referred them to a tertiary-level hospital for further evaluation. Upon evaluating the patient, there was a high level of suspicion for skeletal metastasis being present given the propensity of retinoblastomas to metastasize to the skeleton. With this in mind, it was decided to do a whole-body 18F-fluorodeoxyglucose positron emission tomography-computed tomography (18F-FDG PET-CT) to evaluate the extent of metastatic disease for staging purposes. Unfortunately, the 18F-FDG PET-CT revealed multiple hypermetabolic lesions not only in the skull bones (bilateral frontal and sphenoid bones with epidural extension) but also in the liver (Figure 1).

Based on these findings the child was staged to have a stage IVA2 disease according to the International Retinoblastoma Staging System. A bone marrow biopsy was also done to look for evidence of marrow metastasis, which turned out to be positive for bone marrow infiltration. The child’s prognosis was explained to his parents, and it was decided to start him on high-dose systemic chemotherapy and autologous stem cell transplantation. At the time of writing this case report, the child has been initiated into systemic chemotherapy and is being closely followed up.

## Discussion

Distant metastasis is rare in retinoblastoma and is generally seen in advanced disease. Retinoblastoma metastases have four patterns, each having its own clinical presentation and outcome: trilateral retinoblastoma, regional metastasis, central nervous system (CNS) extension, and distant metastasis [9]. The liver receives an abundant blood supply from both the hepatic artery and portal vein, and this makes it vulnerable to metastasis from distant primary tumors. Imaging methods for diagnosing hepatic metastasis in retinoblastoma include ultrasonography, abdominal computed tomography (CT), and positron emission tomography-computed tomography (PET-CT). The imaging findings on CT can include low-density lesions on CT (due to hypovascularity of the metastases) and intratumoral necrosis. 18F-FDG PET-CT has the advantage of detecting hypermetabolic lesions throughout the body, making it an important tool in accurately staging the disease and consequently helps in prognostication and in the timely institution of adequate treatment in patients. Prognosis remains poor for patients with distant metastasis and high-dose chemotherapy with autologous stem cell rescue has been proven to improve survival in patients with distant metastasis without CNS involvement, like in the case presented here [9,10]. Overall, hepatic metastasis is rare in retinoblastoma and is generally accompanied by skeletal and/or CNS metastases [11,12]. 18F-FDG PET-CT has been shown to play a role in detecting residual/recurrent disease in retinoblastoma patients with locally advanced disease in whom the post-treatment magnetic resonance imaging (MRI) findings were indeterminate [13,14]. 18F-FDG PET-CT does not provide any significant advantage over MRI/CT other than detecting any metastatic lesions that can be missed on conventional imaging [13]. Despite doing a rigorous review of the literature, no case report describing the utility of 18F-FDG PET-CT in detecting hepatic metastasis could be found. This makes this case report all the more valuable from a teaching point of view.

## Conclusions

Retinoblastomas are the most common primary ocular malignancy in children and tends to very rarely metastasize. However, when it does metastasize the prognosis is guarded for these patients. Hepatic metastasis is rare in retinoblastoma and portend a poor prognosis. This case report highlights the importance of PET-CT in detecting distant metastasis in patients with advanced stage disease and may very well be the first case report demonstrating the role of 18F-FDG PET-CT in a retinoblastoma patient with both hepatic and skeletal metastasis.
